# Distractor filtering is affected by local and global distractor probability, emerges very rapidly but is resistant to extinction

**DOI:** 10.3758/s13414-021-02303-3

**Published:** 2021-05-04

**Authors:** Matteo Valsecchi, Massimo Turatto

**Affiliations:** 1 Department of Psychology, University of Bologna, Bologna, Italy; 2 Center for Mind/Brain Sciences (CIMeC), University of Trento, Trento, Italy

**Keywords:** Visual search, Spatial memory

## Abstract

Effects of statistical learning (SL) of distractor location have been shown to persist when the probabilities of distractor occurrence are equalized across different locations in a so-called extinction phase. Here, we asked whether lingering effects of SL are still observed when a true extinction phase, during which the distractor is completely omitted, is implemented. The results showed that, once established, the effects of SL of distractor location do survive the true extinction phase, indicating that the pattern of suppression in the saliency map is encoded in a form of long-lasting memory. Quite unexpectedly, we also found that the amount of filtering implemented at a given location is not only dictated by the specific rate of distractor occurrence at that location, as previously found, but also by the global distractor probability. We therefore suggest that the visual attention system could be more or less (implicitly) prone to suppression as a function of how often the distractor is encountered overall, and that this suppressive bias affects the degree of suppression at the specific distractor-probability location. Finally, our results showed that the effects of SL of distractor location can appear much more rapidly than has been previously documented, requiring a few trials to become manifest. Hence, SL of distractor location appears to have an asymmetrical rate of learning during acquisition and extinction, while the amount of suppression exerted at a specific distractor location is modulated by distractor contextual probabilistic information.

The world provides our cognitive system with a multitude of stimuli which, luckily enough, for the most part do not occur in random fashion. Indeed, the stream of sensory input often presents a certain degree of regularities or covariations, which can be implicitly learned by the human cognitive system to improve performance (Cleeremans et al., 1998). Statistical learning (SL) refers to the ability to extract such statistical structures from the incoming sensory information, a process that requires a repeated exposure to the material, occurs incidentally, and generally does not need awareness or intention to learn (Perruchet & Pacton, 2006).

If on the one hand attention seems to be important for the expression of SL (e.g., Shanks et al., 2005); on the other hand, statistical regularities can affect the deployment of attention as well (Zhao et al., 2013). In particular, in visual search SL facilitates the allocation of attention toward the most likely target location, thus enhancing the efficiency of visual processing (e.g., Chun & Jiang, 1998; Geng & Behrmann, 2005; Miller, 1988; Reder et al., 2003). However, SL also regards irrelevant information, as shown by the fact that through SL, attentional capture by an irrelevant distractor is attenuated. Specifically, evidence suggests that, as a consequence of SL, the location where the distractor appears more frequently would receive a stronger suppression compared to other locations, thus resulting in a reduced priority signal in the saliency map that controls attention, a mechanism whereby visual distraction can be mitigated (e.g., Ferrante et al., 2018; Leber et al., 2016; Sauter et al., 2019; Wang & Theeuwes, 2018; Zhang et al., 2019). Furthermore, SL also attenuates the capture of attention on the basis of color feature regularities, with frequent-color distractors grabbing attention less than infrequent-color distractors (Stilwell et al., 2019).

While SL is an efficient way to extract information from the environment, a key question regards its flexibility, especially because, once established, implicit learning seems to have a strong inertia to adapt to new statistical regularities. For example, when the target occurs more likely in one region of the display, participants implicitly learn this regularity and distribute their attention accordingly, so that the target is detected faster where it is more frequent. This attentional bias can persist for hundreds of trials after the target probability becomes evenly distributed in the display, thus indicating a strong persistence of the original learning (Jiang et al., 2013). Similar findings also have been reported when SL concerns the distractor: Goschy et al. (2014), and Sauter et al. (2019) documented not only that SL of distractor location leads to a local suppression that diminishes attentional capture but also that such learning requires hundreds of trials to be abolished when the probability of the distractor is matched across locations. Analogous lingering effects of SL have been reported by Britton and Anderson (2020), who showed that when in a training phase of 444 trials the distractor appeared more often in one of six possible locations, participants were able to use this statistical regularity to suppress the likely distractor location; however, the statistically learned distractor suppression persisted in the following “extinction” phase of 180 trials, when the distractor location probabilities were equalized. A persistence of the effects of SL of distractor location also has been recently reported by Wang and Theeuwes (2020), as the authors found that SL effects were still present in two extinction blocks of 120 trials each, in which the distractor spatial regularities were removed.

The scenario emerging from this series of studies is that activations in the saliency map induced by SL of distractor location tends to persist for a consistent number of trials after statistical regularities are removed. This reveals a form of SL-induced plasticity of the saliency map that relies on a long-term memory system, and requiring hundreds of trials to be updated. Hence, although this form of learning responds slowly to changes in the statistical distribution of the sensory input, one should also note that the long-lasting effects of SL emerge from studies where the distractor was always present during the so-called extinction phase, during which the distractor probability was made equiprobable at each location (e.g., Britton & Anderson, 2020; Ferrante et al., 2018; Sauter et al., 2019; Wang & Theeuwes, 2020). By contrast, in classical conditioning, from where the notion of extinction originated, the unconditioned stimulus is no longer presented during extinction, which causes the gradual disappearance of the conditioned response (Pavlov, 1927). In the same vein, one may wonder whether the SL-induced inhibition in the saliency map may vanish, or, alternatively, is still retained, in case of a genuine extinction phase, during which the distractor ceases to appear before being reintroduced in the test phase. Perhaps the complete removal of the distractor for a prolonged number of trials might favor the disappearance of the inhibitory effects at the corresponding location in the saliency map, thus abolishing any lingering effects of SL.

A further issue addressed in the present study regards how fast SL of distractor location can be established, as this issue has been previously marginally explored. To gain enough statistical power to detect the early evidence of SL as experience with the distractor unfolds, we analyzed the pooled the data of Block 1 of both Experiment 1 and Experiment 2.

## Experiment 1

In recent years, evidence has accumulated, showing that SL of distractor location is revealed by a reduced capture for the distractor appearing at the most frequent position (see, however, Sauter et al., 2021, for a possible contribution of postselective stage processing), accompanied by a slowing down in target discrimination at the same location. This pattern of results has been proposed to emerge from a suppressive signal applied to the distractor location in the saliency map as a result of the repeated exposure to the distractor spatial regularities (Ferrante et al., 2018; Wang & Theeuwes, 2018). Furthermore, the fact that effects of SL of distractor location persist for a considerable number of trials, after any location bias in the distractor probability has been removed, seems to suggest the existence of a long-lasting memory of the suppressive effects applied to spatial priority map. This memory explains the lingering effects of SL when the spatial distractor probabilities are equalized, during the so-called extinction phase. However, the persistence of the inhibitory effect elicited by the SL phase may have been favored by the fact that the distractor continued to appear during extinction, with the previous most likely location still hosting the distractor in a consistent number of trials, though less often than before. So, the inhibitory effects of SL at a given location could be erased rapidly if the distractor is no longer encountered during a genuine extinction phase, during which any distractor-related processing is halted. Hence, to test whether SL-induced plastic changes in the saliency map can rapidly recover in the absence of a distractor, we first submitted participants to three blocks of trials in which the distractor appeared on 66.6% of all trials. However, when present, the distractor appeared with three different probabilities (60%, 30%, and 10%) in three different locations (training phase); hence, with respect to the total number of trials, the distractor appeared on 40% of trials in the high-probability location, on 20% of trials in the medium-probability location, and on 6.6% of trials in the low-probability location; then, we omitted the distractor for two blocks of trials (extinction phase), which was subsequently reintroduced in the last two blocks, with all locations having the same probability of distractor occurrence (test phase). Crucially, however, whereas in previous studies reporting lingering effects of SL the probability of distractor at each location was equalized at approximately an intermediate level between the previous most and least likely locations, thus maintaining the same overall rate of distractor occurrence, here we decided to make the distractor equiprobable at each location by reducing the previous highest and intermediate probability levels to the lowest probability level (6.6%), which made the distractor appear on 19.8% of all trials. By lowering the overall distractor probability, we increased the chances to observe a consistent and reliable capture effect after extinction in the test phase, and thus possible modulations of capture that may have occurred during extinction in the different distractor locations. This allowed us also to test whether the degree of plasticity in the saliency map is proportional to the amount of inhibition received during SL of distractor location, with more likely distractor locations recovering less from inhibition than other less likely distractor locations. Alternatively, if the previous inhibitory effects accumulated during SL dissipate completely during extinction, then the recovery of capture in the test phase should be the same at each location.

### Method

#### Participants

Due to the lab’s access restrictions dictated by the COVID-19 pandemic situation, experiments were conducted online, and participants were recruited through the Prolific online service (Prolific Academic Ltd, Oxford, UK), with the requirements of being between 18 and 40 years of age, having normal or corrected-to-normal vision, including color vision, and to be running the experiment on a desktop computer. No further information about the observers was obtained by us. We aimed at obtaining 36 data sets, which were complete and with overall accuracy equal or higher than 85%. This required testing a total of 43 participants in Experiment 1.

The sample size was determined using G*Power 3.1.9.7 (Faul et al., 2007) based on the results of a pilot sample of six observers, in relation to the estimated power for the comparison that we deemed to be most crucial to our study. This is the *t* test comparing the capture effect at the high and low probability locations in the test phase (see below), which, if nonsignificant, would have indicated that extinction affects statistical learning. In the pilot data, the *t* test yielded *dz* = 0.4909,^1^ which, in combination with α error probability = 0.05, and power = 0.8, resulted in a sample size estimate of 35 participants. The final sample of 36 was reached due to a slight overestimation of rejected data sets when planning data collection. The same sample size was used in Experiment 2.

All participants were informed about the general aim of the experiment, about their task, and about data-handling procedures in the Prolific interface. They gave their consent by agreeing to be directed to the experiment URL. Observers were paid 7.5 GBP.

All the experiments were carried out in accordance with the Declaration of Helsinki, and with the approval of the local institutional ethics committee (Comitato Etico per la Sperimentazione con l’Essere Umano, Università degli Studi di Trento, Italy).

#### Stimuli and procedure

The experiment was constructed using PsychoPy3 Version 2020.1.3 software (Peirce et al., 2019) and run online using the Pavlovia web hosting service (Open Science Tools Limited, Nottingham, UK). In order to control the retinal size of the stimuli, we asked participants at the beginning of the experiment to position themselves at a distance that was a multiple of a reference distance presented on the screen. Stimuli and procedure were partially based on the additional singleton paradigm described in the seminal study by Theeuwes (1992), and recently adapted to study the relation between SL and attentional capture (Wang & Theeuwes, 2018). Notice that the main effects of SL of distractor location observed with this paradigm were recently shown to be fully replicable also when data were collected via an online experiment (Duncan & Theeuwes, 2020).

**Fig. 1:**
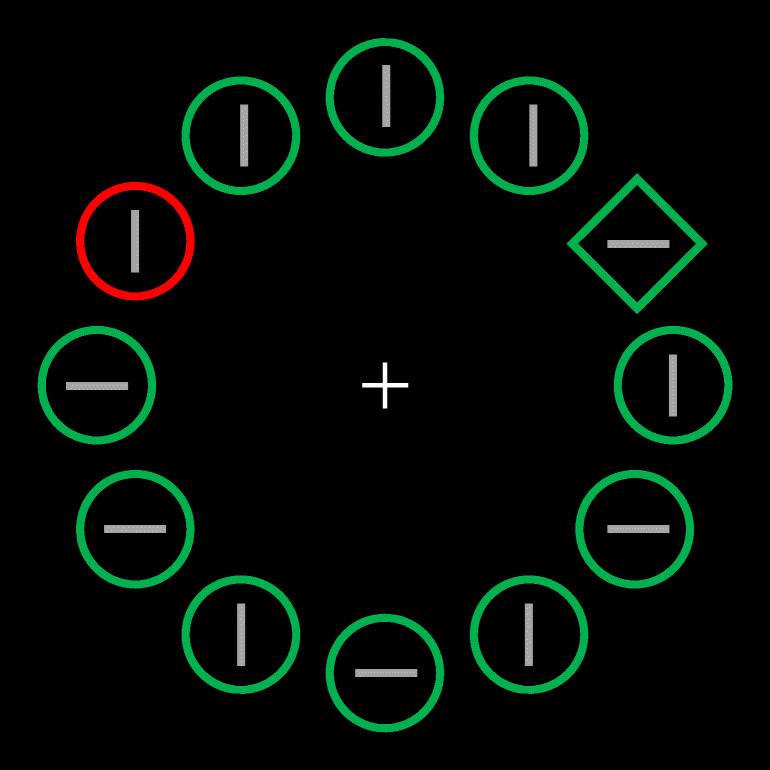
Example of the display used in Experiments 1 and 2. The shape singleton is the target (here, the green diamond), whereas the color singleton is the distractor (here, the red circle). The three allowed distractor locations for such a hypothetical observer would have been at the 2, 6, and 10 o´clock positions. (Color figure online)

Each trial began with the presentation of a white fixation cross at the center of the screen for a random time, uniformly distributed between 0.7 and 1.2 seconds. The fixation point was followed by a target display consisting of 12 shapes (diamonds or circles, colored red or green) arranged on an imaginary ring, each containing a gray bar oriented vertically or horizontally at random (see Fig. 1). Assuming the correct viewing distance, the imaginary circle going through the centers of the shapes had a radius of 3.4° of visual angle. The circle shapes had a diameter of 1.38°, and the sides of the diamond shapes were 1.14° long. The bars inside the shapes were 0.55° long.

On each trial, the target was the shape singleton (either the circle among diamonds or vice versa, randomized across trials), whereas when present the distractor was the color singleton (either red or green, randomized across trials). When no distractor was presented, all shapes had the same color (red or green, randomized across trials). The participants’ task was to press, as quickly as possible, the keys “v” or “h” of the keyboard to indicate whether the gray bar inside the target was vertical or horizontal, and to ignore the color distractor if present. The display remained on the screen until participants responded. The next trial started 2 seconds after the response was given. Response times (RTs) were recorded from the target appearance, and in case the response was incorrect, the message “Wrong!,” in red letters, was presented for 300 ms during the intertrial interval.

Although the display consisted of 12 elements, the target and the distractor could appear only at three equidistant locations (e.g., at 2, 6 and 10 o´clock positions), which were chosen randomly for each participant among the four possible configurations. These locations were further assigned randomly for each participant to be the high, medium, and low probability distractor locations, whereas the remaining locations were occupied by nontarget elements.

#### Experimental design

The experiment consisted of a training and a test phase, separated by an extinction phase. Participants were administered with three blocks of 105 trials during training, two blocks of 55 trials during extinction, and two blocks of 105 trials during test (635 trials in total), and were allowed to take a brief rest between blocks. In the training phase, each block was made of seven miniblocks of 15 trials each, with and five distractor-absent trials and 10 distractor-present trials subdivided as follows: six in the high-probability distractor location (40% of the total trials), three in the medium-probability distractor location (20% of the total trials), and one trial in the low-probability distractor location (6.6% of the total trials). The test phase also consisted of seven miniblocks of 15 trials, which were now divided into 12 distractor-absent trials and three distractor-present trials per miniblock. Thus, the distractor appeared in one trial per miniblock at each location—namely, we equalized the probability of distractor occurrence to the lowest level of the training phase (6.6%) at each location. Finally, no distractor was presented in the two blocks of the extinction phase.

Before beginning the experiment, participants performed a single miniblock of 15 trials, the same as those of the training phase, to familiarize themselves with the task.

#### RT analysis

The analysis of RT data collected outside of a laboratory setting required particular care in the removal of outliers. To this aim, we followed a two-step procedure. First of all, for each participant and cell of the experimental design we applied an outlier removal procedure based on median absolute deviation (MAD; Leys et al., 2013). The threshold we decided to use was equal to three MADs. In the second step, in order to further reject implausible values, we discarded all remaining RTs higher than 2,500 and lower than 200 ms., which led to an overall proportion of discarded trials of 9.5%. Data analyses and calculations for outlier removal were only based on correct trials.

In order to ensure that the effects of the probability of distractor occurrence at a given location were due to the probability itself and not to short-range intertrial priming effects, we removed from the analysis all the trials where the distractor appeared in the same position of the preceding trial. Unless specifically indicated, including or excluding repeated location distractor trials had no effect on the overall pattern of results.

## Results and discussion

**Fig. 2:**
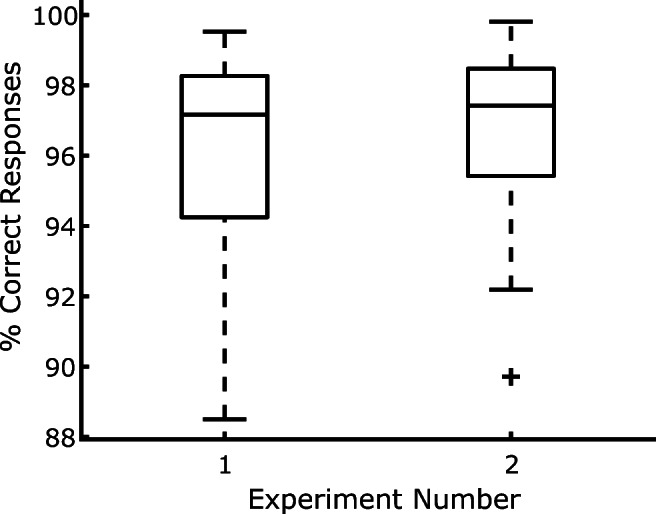
Boxplots of individual response accuracies for the participants in the two experiments

As depicted in Fig. 2, for participants whose data were not discarded the percentage of correct responses was generally high (>95%), and therefore accuracy was not further analyzed.

**Fig. 3:**
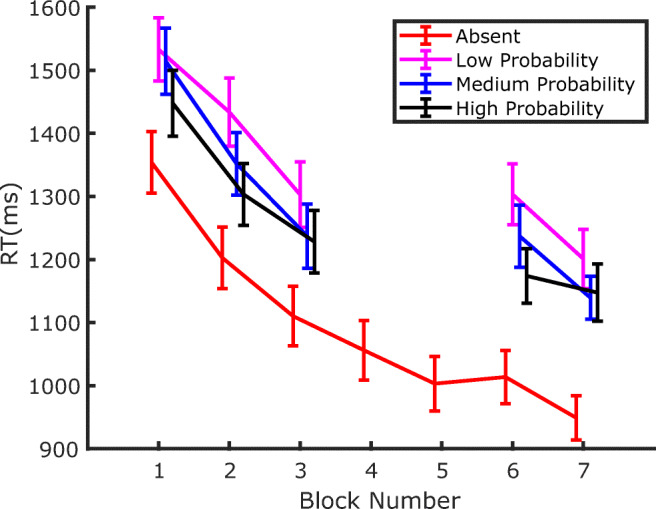
RTs in Experiment 1 as a function of block number. Separate lines identify the trials where the distractor was either absent or appeared with high, medium, or low probability, in the corresponding location. Error bars are standard errors of the mean (*SEM*). Notice that Blocks 4 and 5 constituted the Extinction phase, where no distractors were presented

RTs for correct responses from all blocks of Experiment 1 are depicted in Fig. 3. A few observations seem quite evident: first, participants became substantially faster throughout the whole experiment; second, RTs are lengthened by the presence of the distractor (both before and after extinction); third, the detrimental effect of the distractor on RTs decreases as the probability of distractor occurrence at a given location increases. In other words, and in agreement with previous findings (Wang & Theeuwes, 2018), distraction is attenuated at locations where the distractor is more likely to occur.

**Fig. 4:**
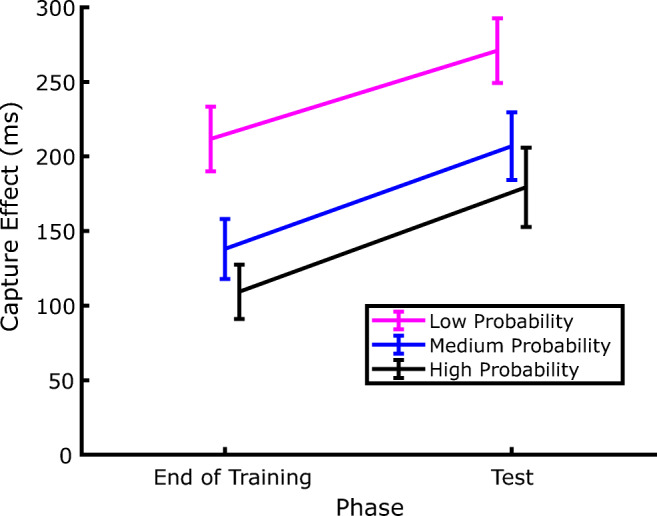
Evolution of the capture effect across the extinction phase. The capture effect is computed as the difference between the RTs of trials where the distractor was presented at a given location and the trials where the distractor was absent, in the corresponding phase of the experiment. The data labelled as End of Training are from Blocks 2 and 3, whereas the Test trials include both Blocks 6 and 7. Error bars represent *SEM*s

Since the aim of the experiment was to address whether SL of distractor location persists even during a genuine extinction phase, our statistical analyses focused on how the amount of capture (defined as the RT difference between distractor-present and distractor-absent trials) changed between the last two blocks of the training phase and the two blocks of the test phase.^2^ The corresponding data are presented in Fig. 4, which shows that, as expected, by the end of the training phase attentional capture was modulated by the distractor probability associated with the different locations. Interestingly, since the same pattern of capture was evident also in the test phase, it is evident that the SL-induced attentional modulation persisted during the extinction phase despite the distractor being completely omitted. This confirms that, once established, SL of distractor location also may have a strong inertia to change when no distractor is experienced during the extinction phase. Notably, the effects of SL on attentional capture in the test phase were evident despite the fact that the distractor probability was equalized to the lowest probability at all locations. These observations were supported by a within-participants repeated-measures analysis of variance (ANOVA), with phase (training vs. test) and distractor probability (low, medium, or high) as factors. The results revealed significant effects of both phase, *F*(1, 35) = 20.215, *p* < .001, *η*_*p*_^*2*^ = .366, and probability, *F*(2, 70) = 11.615, *p* < .001, *η*_*p*_^*2*^ = .249, and no significant interaction, *F*(2, 70) = .072, *p* = .93, *η*_*p*_^*2*^ = .002. Subsequent pairwise comparisons (one-tailed *t* tests) showed that the amount of capture in the high-probability distractor location was smaller compared with the low-probability distractor location, in both the training phase, *t*(35) = 4.155, *p* < .001, *d* = .692, and the test phase, *t*(35) = 3.168, *p* = .002, *d* = .528.

Additionally, as shown in Fig. 4, the capture effect increased systematically after extinction irrespective of distractor probability, as attested by the lack of a significant Phase × Probability interaction. This result might be expected for the high-probability and medium-probability distractor locations, as in both cases the distractor probability diminished to the lowest level in the test phase, which reasonably produced an increase of capture, high-probability distractor location, *t*(35) = 2.872, *p* = .007, *d* = .478; medium-probability distractor location, *t*(35) = 3.452, *p* = .001, *d* = .575. However, such capture increment emerged also in the low-probability distractor location, where in fact the rate of distractor occurrence did not change between the training and test phases. Hence, despite that the distractor probability was the same (6.6%), the attentional capture was stronger in the test phase as compared with the training phase, *t*(35) = 2.325, *p* < .026, *d* = .387.

**Fig. 5:**
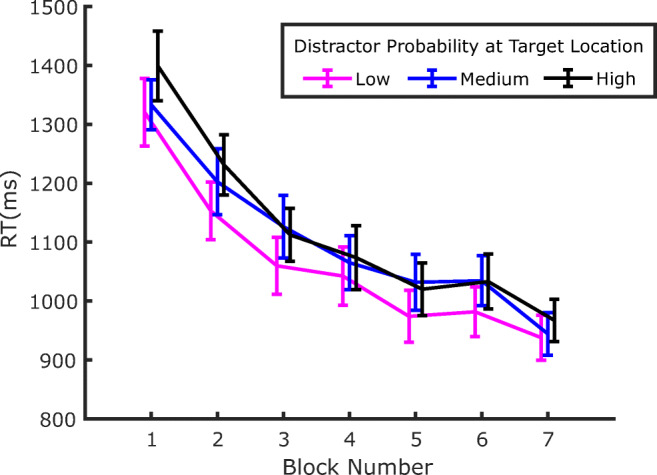
Evolution of RTs in distractor absent trials plotted as a function of the target location. Notice that the pattern of results seems to be reversed relative to Fig. 3, indicating that reduced distraction at a given location implies reduced responsiveness to the target as well. Error bars represent *SEM*s

As a final analysis, we also investigated whether SL also affects target processing in distractor-absent trials—namely, the target-location effect. If the reduction of distraction at the high-probability distractor location is due to a relatively higher suppression of that location, then target processing should also be slowed down at that location compared with other locations (Wang & Theeuwes, 2018). The relevant data are plotted in Fig. 5. At least qualitatively, the data seem to show the expected pattern (i.e., the reverse ordering of results relative to Fig. 3, with longer RTs at the high-distractor probability location relative to the low-distractor probability location). This also seems to be the case in the training, extinction, and test phases. In order to evaluate the effect of target location statistically, we submitted the results to three separate ANOVAs, with target location as factor (low, medium, or high distractor probability), one for each phase: training (Blocks 2–3), extinction (Blocks 4–5), and test (Blocks 6–7). The effect of target location was significant in the training phase, *F*(2, 70) = 5.209, *p* = .008, *η*_*p*_^*2*^ = .129, but, although it remained numerically similar, it failed to reach significance in the extinction phase, *F*(2, 70) = 2.279, *p* = .109, *η*_*p*_^*2*^ = .061, and in the test phase, *F*(2, 70) = 2.180, *p* = .12, *η*_*p*_^*2*^ = .058.

In sum, the two the main findings of Experiment 1 can be summarized as follows: first, SL of distractor location does not dissipate during a true extinction phase lasting more than 100 trials, indicating that a memory of the level of suppression at the specific distractor location, which is proportional to its probability, is maintained even when the distractor is not encountered for many trials in the extinction phase. This in turn reveals a long-lasting memory of the suppressive signals dictated by SL, which operates on the saliency map that controls attention. The analysis of RTs on distractor-absent trials—namely, the target-location effect—seems to be somewhat inconclusive with respect to this point. While RTs still tend to be shorter in the extinction phase when targets are presented at the low-distractor probability location, the result did not reach significance, which might have to do with the fact that the target-location effect tends to be less evident than the distractor-location effect, especially when the local probabilities of distractor occurrence are not extremely different (Lin et al., 2020).

Second, when the distractor was reintroduced in the test phase, the amount of capture increased at all locations, including the low-probability location, where the distractor probability was the same as the training phase. A straightforward explanation of this pattern of results is that the amount of capture increased at each location, compared to the end of training, because the distractor was completely omitted during extinction. In other words, during extinction, the display acted as a cue for the distractor absence, so that participants learned to expect a display without the distractor, an expectation that accrued also at the low-probability location, and that was violated when the distractor reappeared in the test phase. However, a more intriguing possibility is that the level of suppression at a given location does not depend solely on the specific probability of distractor occurrence at that location, as previously reported (Ferrante et al., 2018; Wang & Theeuwes, 2018), but is affected also by the overall distractor probability. Accordingly, when after the extinction phase the distractor was reintroduced with the same low-level probability at each location (6.6%), the overall level of distractor probability diminished from 66.6% in the training phase to 19.8% in the test phase, which may have attenuated the strength of the suppressive signals at each location. The next experiment was conducted with the aim of clarifying which explanation better accounts for the results.

## Experiment 2

In order to understand the reason for the increased capture at the low-probability location observed in the previous experiment, we decided to remove the extinction phase. We reasoned that if the observed effect were due the unexpected reappearance of the distractor after the extinction phase, then no increase of capture for the low-probability distractor location should be found when such phase is removed. Note that, according to this hypothesis, an increase of capture for the other two locations should still be observed, because in both locations the probability of the distractor drastically decreased with respect to the training phase. By contrast, if the reason for the increased capture at the low-probability distractor location were due to a mechanism that modulates the level of capture by computing not only the local distractor probability but also the global distractor probability, then we should expect the same pattern of results observed in Experiment 1—namely, capture should also increase at the low-probability distractor location.

### Method

#### Participants

Participants were recruited following the same procedure and the same criteria as in Experiment 1. Obtaining 36 data sets with average accuracy above 85% required testing a total of 42 participants. They were compensated with 6.75 GBP for participation, amounting to approximately the same hourly fee as for Experiment 1, given the shorter duration of Experiment 2.

#### Stimuli and procedure

Stimuli and procedure were the same as in Experiment 1. The experimental design for Experiment 2 was identical to that of Experiment 1, with the exception that no extinction blocks were administered (for 525 trials in total). This implies that between the third and the fourth block, the distractor probability became instantly identical in all three locations, while the overall distractor probability decreased from 66.6% to 19.8%.

#### RT analysis

RT outliers were identified with the same algorithm used in Experiment 1. The overall proportion of discarded trials was 10.4%.

### Results and discussion

As depicted in Fig. 2, for participants whose data were not discarded, accuracy was generally high (>95%), and therefore it was not analyzed further.

**Fig. 6:**
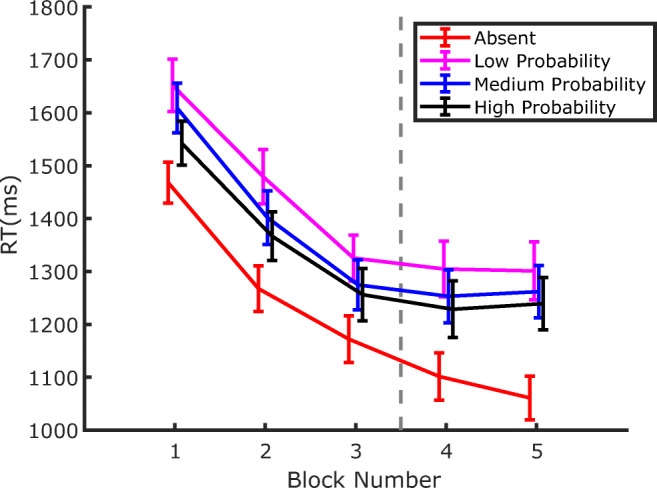
RTs in Experiment 2 as a function of block number. Separate lines identify the trials where the distractor was either absent, or in one of the three possible locations. Error bars are standard errors of the mean (*SEM*). The vertical dashed line marks the boundary between the training phase, where distractors were presented according to the probability associated with the location (Blocks 1–3), and the test phase, where distractor probability became equal to the one of the low-probability distractor location in all three locations (Blocks 4–5)

RTs from Experiment 2 are shown in Fig. 6. The pattern of results appears very similar to the one observed in Experiment 1: first, participants became substantially faster throughout the whole experiment; second, RTs were lengthened by the distractor presence, both in the training phase, where the distractor probability varied between locations, and in the test phase, where it was equalized at the lowest probability level at all locations; third, during training, attentional capture was modulated by the distractor probability, an effect that persisted during the test phase.

**Fig. 7:**
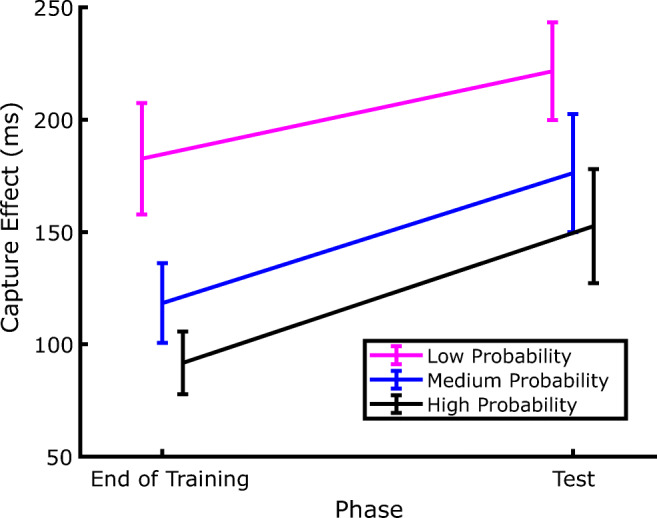
Evolution of the capture effect after the distractor probability became low at all locations. The capture effect is computed as the difference between the RTs of trials where the distractor was presented at a given location and the trials where the distractor was absent, in the corresponding phase of the experiment. Notice that the location definitions pertain to the training phase, because in the final phase the probability of occurrence of the distractor was low in all three locations. The End of the Training data are from Blocks 3 and 4, whereas the Test trials include both Blocks 4 and 5. Error bars represent *SEM*s

Figure 7 depicts the amount of capture as a function of distractor probability at the end of the training phase (Blocks 2 and 3) and in the test phase (Blocks 4 and 5). The results show a pattern very similar to that observed in Experiment 1, with larger capture for less probable distractor in both the training and test phase. This was confirmed by a repeated-measures ANOVA, with phase (training vs. test) and distractor probability (low, medium, or high) as factors. The results revealed significant effects of both phase *F*(1, 35) = 10.128, *p* = .003, *η*_*p*_^*2*^ = .224, and probability *F*(2, 70) = 8.081, *p* < .001, *η*_*p*_^*2*^ = .187. As in Experiment 1, no significant interaction emerged between phase an probability, *F*(2, 70) = 0.212, *p* = .809, *η*_*p*_^*2*^ = .006, indicating that the amount of capture increased significantly regardless of distractor probability when this was set at the lowest level at all locations (see Fig. 7). Indeed, pairwise comparisons (one-tailed *t* tests) indicated that capture increased at the medium-probability distractor location, *t*(35) = 2.39, *p* = .011, *d* = .398, and at the high-probability distractor location, *t*(35) = 2.363, *p* < .011, *d* = .393, where the local distractor probability decreased in the test phase. The RT pattern in the case of the low-probability distractor location seems to be compatible with the finding of Experiment 1, showing an increase of 60 ms in the capture effect as the overall probability of distractors decreases while local probability remains the same, although this result was not statistically significant *t*(35) = 1.292, *p* = .102, *d* = .215.^3^

**Fig. 8:**
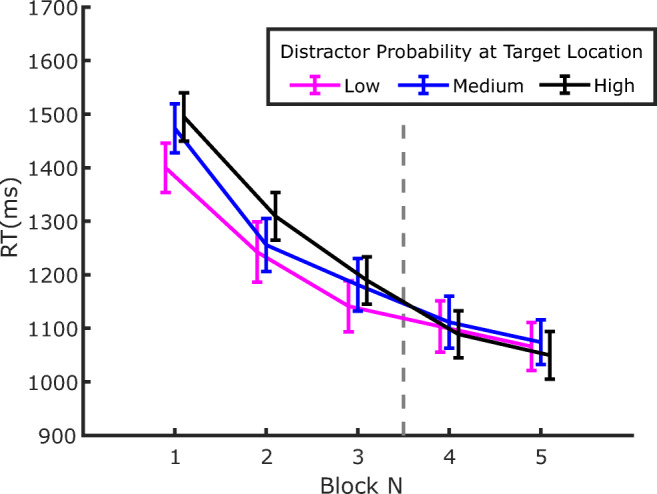
Evolution of RTs in distractor-absent trials plotted as a function of the target location. Notice that the pattern of results in the first three training blocks seems to be reversed relative to Fig. 6, indicating that reduced distraction at a given location implies reduced responsiveness to the target as well. The effect seems to dissipate immediately in the following test blocks. Error bars represent *SEM*s. The vertical dashed line marks the boundary between the training phase, where distractors were presented according to the probability associated with the location (Blocks 1–3), and the test phase where distractor probability became equal to the one of the low probability location in all three locations (Blocks 4–5)

As a final step, we investigated whether a target-location effect emerged in the present experiment in distractor-absent trials. The relevant data are plotted in Fig. 8. Contrary to Experiment 1, here it appears that the modulation of RTs as a function of the target location, which is already relatively weak in the training phase, disappears immediately after the distractor probabilities are equalized. In order to evaluate this effect statistically, we submitted the results to two separate ANOVAs, with target location as a factor (low, medium, or high distractor probability), one for each phase: training (Blocks 2–3) and test (Blocks 4–5). The effect of target location reached significance neither in the training phase, *F*(2, 70) = 1.813, *p* = .171, *η*_*p*_^*2*^ = .049, nor in the test phase, *F*(2, 70) = 0.557, *p* = .575, *η*_*p*_^*2*^ = .016.

### Combined analysis of Block 1 from both experiments

If one accepts the idea that attentional capture is modulated by SL of distractor location, then such a process will inevitably require a minimum amount of experience with the distractor in order to estimate its probability of occurrence at a given location. Hence, an interesting question concerns the speed of SL acquisition—namely, how many trials are necessary before SL can manifest its effects on capture. Previous works have only marginally addressed this issue, so as to show SL of distractor location RTs are generally pooled together across the different blocks of exposure to the distractor (e.g., Wang & Theeuwes, 2018). In fact, an attempt has been made by Ferrante and colleagues to characterize the temporal development of SL of distractor location, showing that it took approximately 300 trials and 150 trials to become manifest in their Experiments 2 and 3, respectively (Ferrante et al., 2018). However, since a close inspection of Figs. 3 and 6 indicates that, in the present study, the dependency of capture on the distractor probability at a given location is already well established in the first block of both Experiment 1 and Experiment 2, we decided to address this issue in more detail. To investigate how early this effect emerges within the first block of trials, in the following analyses we pooled together the data of the two experiments. Notice that this is also justified by the fact that the experimental procedure was identical in the two experiments up to the end of the training phase (Block 3).

As we anticipated in the Methods section, Block 1 consisted of seven repetitions of miniblocks of 15 trials, each containing five trials where the distractor was absent, and six, three, and one trials where it appeared at the high, medium, and low probability location, respectively. We therefore progressively aggregated the data from the seven repetitions to investigate at what point the location-probability effect emerged. Notice that for the low-probability distractor location, each repetition corresponds to a single trial, so, for this analysis, we only discarded outlier RTs based on the fixed 200 and 2,500 ms because it is not possible to estimate the MAD of RTs and use the adaptive algorithm. Furthermore, we only retained the data from participants that had at least one correct trial in all the cells of the design within the data interval considered. Also, notice that before the beginning of the experiment participants underwent one miniblock of 15 trials of practice with the same proportion of distractors at the different locations. Practice trials were not included in the analysis due to the high error rate and very long RTs, but, still, it must be considered to determine how fast SL can develop.

**Fig. 9:**
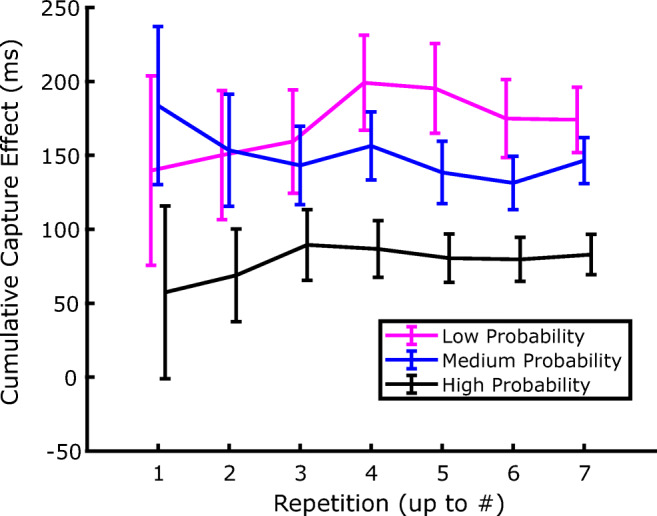
Evolution of the cumulative capture effect in the first block of Experiment 1. Data are plotted relative to Repetition 1, Repetitions 1+2, Repetitions 1+2+3, and so on, for distractors occurring at the low, medium, and high probability locations. Error bars are *SEM*

The average RTs resulting from this aggregation procedure are presented in Fig. 9. Although the expected separation between the RTs based on the distractor location is already numerically present when the first repetition is considered in isolation, it is also evident that there is considerably high interindividual variability. We therefore decided to submit the data of the cumulative capture effect to sequential one-way repeated-measures ANOVAs, with location (low vs. medium vs. high probability) as a factor. As Table 1 shows, the test begins to be significant when four repetitions (i.e., 60 trials in total, 40 distractor-present and 20 distractor-absent trials) are cumulated, corresponding to 75 trials of experience with the distractor contingencies including the practice miniblock. If trials with distractors repeated at the same location were not removed, the SL effect would be significant already when three repetitions are cumulated.

**Table 1:** Summary of the results of the sequential ANOVAS performed on the cumulative aggregated data of Block 1 from both experiments

Cumulated Repetition	*N*	*F*	*df*	*p*	*η*_*p*_^*2*^
1	46	1.542	2, 90	.22	.033
2	59	2.202	2, 116	.115	.037
3	67	1.835	2, 132	.164	.027
4	71	5.783	2, 140	.004	.076
5	72	7.231	2, 142	<.001	.092
6	72	7.234	2, 142	<.001	.092
7	72	9.873	2, 142	<.001	.122

*Note.* Notice that the *N* increases as the number of cumulated repetitions increases, due to the fact that less and less participants have to be discarded due to missing data.

## Experiment 3

Experiments 1 and 2 provided inconclusive results regarding the question of whether the inhibition of the frequent distractor location continued to manifest itself in a relative increase of RTs when the target appeared at that location once the distractor contingencies were removed (i.e., in the extinction phase of Experiment 1 and in the test phase of Experiment 2). We speculated that one possible reason for this could be that the RT cost for targets presented at the frequent distractor location tends to be reliable only when the ratio of distractor probability between target and distractor locations is relatively high (Lin et al., 2020). In particular, Lin et al. (2020) only observed a reliable effect in distractor-absent trials when the distractor was 8 times more likely to appear at the high-probability distractor location compared to the low-probability distractor location. In the training phases of Experiments 1 and 2 of the present study, the distractor was only 6 times more likely to appear at the high-probability location, as compared with the low-probability location, a ratio that very likely was not large enough to produce a reliable effect in distractor-absent trials, as also attested by Lin et al. (2020). Hence, in Experiment 3, we decided to modify the experimental design of Experiment 1 to increase the ratio of distractor probability between the high and low probability locations in the training phase, in the attempt to verify whether this produced a reliable effect of target position in the extinction phase.

### Method

#### Participants

Participants were recruited following the same procedure and the same criteria as in Experiment 1. In this case, we aimed at obtaining 18 data sets with average accuracy above 85% (see below), which required testing a total of 24 participants. They were compensated with 7.5 GBP for participation, for a duration of the experiment exactly equivalent to the one of Experiment 1.

Since the main reason for conducting the experiment related to the target position effect in the extinction phase, we established the sample size based on the comparison between RTs when targets appeared at the high-probability distractor location and at the low-probability distractor location in the extinction phase (Blocks 4 and 5 combined). After testing a pilot sample of 10 observers, we determined the sample size using G*Power 3.1.9.7 (Faul et al., 2007). In the pilot data, the critical *t* test yielded *dz* = 1.723, which, in combination with α error probability = 0.05, and power = 0.8, resulted in a sample size estimate of seven participants. Despite the fact that the power analysis confirmed that our sample size was already adequate, we still decided to increase it to 18 observers, so as to reach at least half the sample size that was used in the first two experiments.

#### Stimuli and procedure

The stimuli and procedure were the same as in Experiment 1. The experimental design for Experiment 3 was similar to that of Experiment 1, with three blocks of 105 trials during training, two blocks of 55 trials during extinction, and two blocks of 105 trials during test (635 trials in total). Crucially however, instead of defining a low-probability, a medium-probability, and a high-probability distractor location, there were one high-probability distractor location and two low-probability distractor locations (all equidistant from each other). Due to the need to equalize the probabilities, each training or test block was run as a fully randomized series of 105 trials (i.e., there was no further division in miniblocks). In each block, the distractor appeared three times at each low-probability location, 64 times at the high-probability location, and was absent in 33 trials. This gave an overall probability of distractor occurrence in the training phase of 66.6% of the total trials, an overall probability of occurrence of 60.9%, and 2.9% at the high-probability and low-probability locations, respectively. This meant also that the distractor was 21.3 times more likely to occur at the high-probability location compared with each low-probability location, which we expected to be sufficient to generate a reliable effect of target position in distractor-absent trials. In the test phase, we equalized the probability of distractor occurrence at each location to the level of the low probability in the training phase (i.e., three trials per location per block of 105 trials). This is equivalent to a general distractor probability of 8.6% and a local distractor probability of 2.9%.

#### RT analysis

RT outliers were identified with the same algorithm used in Experiment 1. The overall proportion of discarded trials was 10.9%. Notice that the data from the two low-distractor probability locations were pooled together in all the analyses of Experiment 3.

### Results and discussion

The overall accuracy in the observers that were included in the sample was on average 95.8%, with all individual values above 90%, and was not further analyzed.

**Fig. 10:**
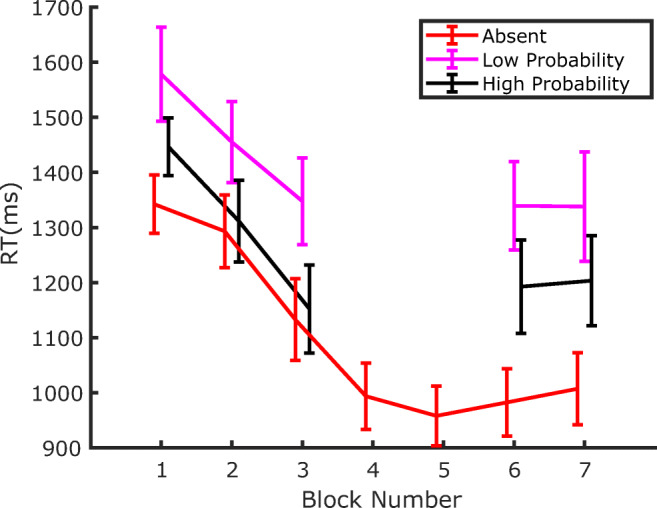
RTs in Experiment 3 as a function of block number. Separate lines identify the trials where the distractor was either absent, or appeared at the high-probability or low-probability location. Error bars are standard errors of the mean (*SEM*). Notice that Blocks 4 and 5 constituted the extinction phase, where no distractors were presented

The average RTs from Experiment 3 are depicted in Fig. 10. The results seem, broadly speaking, compatible with those of Experiments 1 and 2, with the possible difference that RTs in the high-probability distractor location seem closer to RTs in the distractor-absent condition, which would be consistent with a stronger effect of distractor location as a consequence of the more extreme probability difference. Notice that the relative RT advantage for responding to targets when the distractor appeared in the high-probability location still seems to be present in the test phase when the actual distractor probability was the same across all locations, as we observed in the first two experiments.

**Fig. 11:**
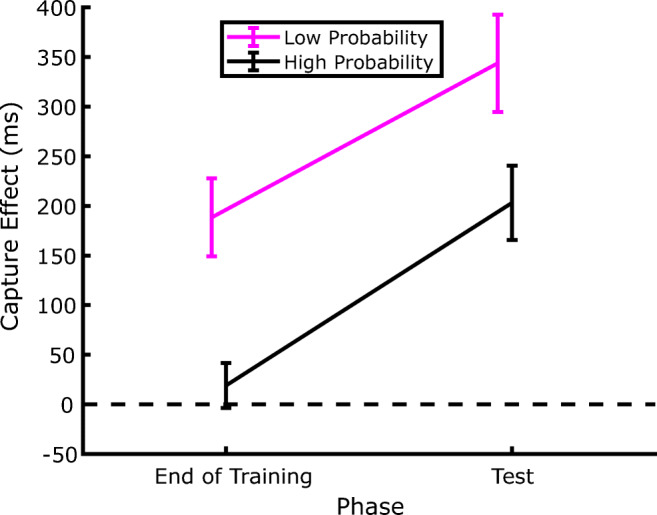
Evolution of the capture effect across the extinction phase in Experiment 3. The capture effect is computed as the difference between the RTs in trials where the distractor was presented at a given location and the trials where the distractor was absent, in the corresponding phase of the experiment. The data labelled as End of Training are from Blocks 2 and 3, whereas the Test trials include both Blocks 6 and 7. Error bars represent *SEM*s

In order to investigate statistically the reliability of the effects of distractor location on RTs, we further analyzed the data by comparing the capture effects (i.e., the difference between RTs in distractor-present and distractor-absent trials) at the end of the training phase (Blocks 2 and 3) with those from the test phase (Blocks 6 and 7). The corresponding average results are plotted in Fig. 11.

First of all, we conducted a within-participants repeated-measures ANOVA, with phase (training vs. test) and distractor probability (low vs. high) as factors. The results revealed significant effects of both phase *F*(1, 17) = 26.525, *p* < .001, *η*_*p*_^*2*^= .609, and probability *F*(1, 17) = 13.115, *p* = .002, *η*_*p*_^*2*^ = .435, and no significant interaction, *F*(1, 17) = .127, *p* = .724, *η*_*p*_^*2*^ = .007. Subsequent pairwise comparisons (one-tailed *t* tests) showed that the amount of capture in the high-probability distractor location was smaller compared with the low-probability distractor location, in both the training phase, *t*(17) = 3.381, *p* < .003, *d* = .797, and the test phase, *t*(17) = 2.123, *p* = .048, *d* =.501.

The main effect of phase in the ANOVA, in the absence of a significant two-way interaction, indicates that, coherently with what we found in Experiment 1, the capture effect increased systematically after extinction irrespective of the actual distractor probability. Once again, direct comparisons showed that the increase in capture between the training and the test phase was significant both at the high-probability distractor location *t*(17) = 2.473, *p* < .024, *d* = .583, where it could be explained by the decrease of distractor probability, but also in the low-probability distractor locations, *t*(17) = 4.827, *p* < .001, *d* = 1.137, where the local distractor location probability did not change. This confirms the finding that the amount of capture is modulated not only by the local but also by the global distractor probability.

**Fig. 12:**
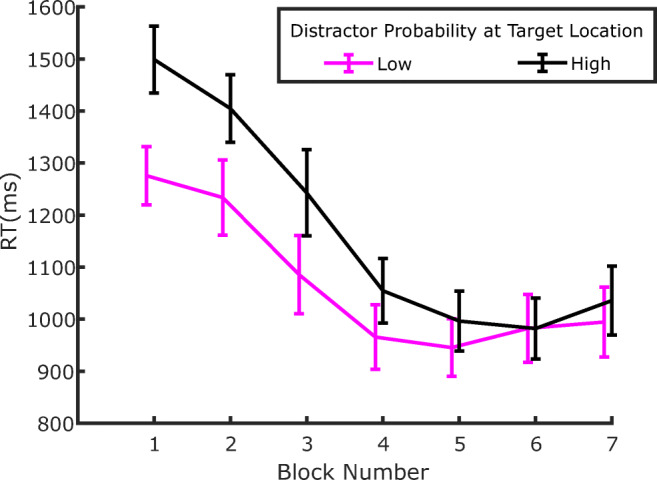
Evolution of RTs in distractor absent trials plotted as a function of the target location. Notice that in the training (1–3) and extinction blocks (4–5), RTs are higher at location that has the highest distractor probability, indicating that reduced distraction at a given location implies reduced responsiveness to the target as well. The effect vanishes in by the beginning of the testing phase. Error bars represent *SEM*s

Having verified that all the relevant findings concerning the effects of distractor position from the previous experiments are replicated in Experiment 3, we evaluated the effect of target position in distractor-absent trials, which was the main reason for conducting the experiment. The relevant data are plotted in Fig. 12. Evidently, as a result of the more extreme distractor probability ratio between the high and low condition, the lengthening of RTs to targets presented at the high-probability distractor locations was very pronounced in the training phase. This effect seemed also to extend into the extinction phase, whereas by Block 6 (i.e., at the beginning of the test phase), it appeared to have vanished. We performed three paired *t* tests, one for each phase, to verify these observations statistically. The comparisons were significant in the training (Blocks 2–3), *t*(17) = 3.959, *p* = .001, *d* = .933, and extinction (Blocks 4–5), *t*(17) = 3.287, *p* = .004, *d* = .774, phases, but not in the test phase (Blocks 6–7), *t*(17) = 0.781, *p* = .445, *d* = .184.

## General discussion

The results of the present study suggest three main conclusions: first, lingering inhibitory effects at distractor location due to SL are also observed when the distractor is completely removed during a genuine extinction phase; second, the level of suppression exerted at a given distractor location via SL does not depend solely on the distractor probability at the specific location, but is affected also by the overall distractor probability; third, given the appropriate conditions the effects of SL of distractor location can appear very rapidly, requiring a few trials to become manifest.

SL of distractor location attests the capacity of the visual system to extract regularities in the sensory input concerning the occurrence of salient but irrelevant stimuli, and to adapt the filtering processing accordingly (Ferrante et al., 2018). Hence, an interesting question regards the flexibility of such learning mechanism in adapting to changes in the stimulating conditions. Previous studies have addressed this issue by removing the distractor-location contingencies used during training, and by testing how rapidly the system readjusts the pattern of distractor suppression across the different locations. However, in these studies the so-called extinction phase actually consisted of a series of trials where the distractor, after training, appeared equally often at all locations, thus removing previous unbalances in the rate of distractor occurrence (e.g., Britton & Anderson, 2020; Ferrante et al., 2018; Sauter et al., 2019; Wang & Theeuwes, 2020). Quite consistently, these studies showed that once the effects of SL of distractor location are established, they tend to persist for hundreds of trials after the distractor probability is equalized at all locations. However, we reasoned that the presence of such lingering effects of SL may have been favored by the fact that the distractor was still presented during the extinction phase, which does not represent the most stringent test to evaluate the persistence of SL effects. Conversely, the removal of the distractor in a true extinction phase would have given no incentive to the cognitive system for maintaining the previous pattern of distractor suppression at different locations, so that we could evaluate whether the system rapidly learns to adapt to a new situation when no suppressive signals are required to protect target processing from interference. In Experiment 1, we therefore inserted a true extinction phase, during which no distractor was presented between the training and the test phase. The results showed that SL of distractor location survived the true extinction phase, as attested by the different RTs observed in the high, medium, and low probability location in the test phase. These findings suggest that the modulation of the saliency map exerted via SL of distractor location during training was not abolished despite the distractor removal in the extinction phase, thus revealing that the pattern of activations in the saliency map is maintained in a relatively long-term memory system, which is not immediately updated even when the distractor is not encountered for many trials (here, 110 trials). In agreement with this view, in Experiment 3 we also documented a reliable effect of distractor location on target processing that persisted during the extinction phase, with the target being discriminated more slowly at the previous high-probability distractor location than at the low-probability distractor location (Wang & Theeuwes, 2018; Zhang et al., 2019).

The long-lasting effects of SL of distractor suppression documented in previous studies and in the present one might seem at odds with recent findings indicating that the spatial distribution of suppression achieved via SL can be remarkably flexible, adapting to changes in the distractor statistical regularities in space (Wang & Theeuwes, 2020). It should be noted, however, that in the study of Wang and Theeuwes (2020), the distractor occurred at a different high-probable location in each of the three consecutive sessions, consisting of 240, 480, and 480 trials, respectively. Hence, the high-probable distractor location changed two times in the experiment, at the end of the first session (i.e., after 240 trials), and at the end of the second session (i.e., after 480 trials). In other words, in each session participants were exposed to a significant number of trials during which participants had plenty of occasions to learn the new distractor location. In addition, one may also note that the difference between the flexibility of SL documented by Wang and Theeuwes (2020), and the seeming lack of it in previous studies (and in the present one) reporting a suppressive bias that persists when the distractor statistical regularities are no longer in place (e.g., Ferrante et al., 2018; Wang & Theeuwes, 2020), might in fact be more apparent than real. Actually, changing the previous distractor spatial regularities by introducing new regularities might not be equivalent to removing such regularities. In the former case, failing to adjust the pattern of suppression to the new spatial contingencies would inevitably make the attentional system more vulnerable to distraction. By contrast, to maintain the previous suppressive bias when, in fact, the distractor is equally likely to occur at each location might not be particularly harmful for the attentional system. Indeed, what may be lost in terms of filtering efficacy at locations suppressed less than what would be suggested by the new distractor probability distribution is gained at the location that is suppressed more than what would be necessary. In other words, the negative consequences of maintaining the previous suppressive bias when the probabilities of distractor occurrence are equalized across locations are much less severe than to maintain such bias when the high-probability distractor location changes. This might explain why the mechanism based on SL of distractor location would seem to be more flexible when new contingencies are introduced than when previous contingencies are removed.

Another possible explanation for the observation that statistical learning of distractor location is established quickly and flexibly, and at the same time it is stable and resilient to extinction, is that two storage systems are involved in the control of capture suppression based on the previous history of distractor occurrence, a short-term memory system and a long-term memory system. A similar idea has been proposed to explain how the habituation of the startle response is established (Davis, 1970). In the case of distractor suppression, the short-term memory system would be responsible for the almost instantaneous buildup of local inhibition at likely distractor locations, whereas the long-term memory system would be responsible for its stability over longer time periods. Notice that the fact that humans can learn both quickly and stably is clearly established in the case of sensorimotor contingencies—for instance, in the case of saccade adaptation (e.g., McLaughlin, 1967) or transsaccadic perceptual recalibration (Valsecchi et al., 2020). Accordingly, it has been suggested that the capacity to learn at different time scales is a general property of the nervous system, from low-level sensorimotor learning, to higher-level cognitive learning (Kording et al., 2007).

The second relevant (and quite unexpected) finding of our study is that distractor suppression at a specific location is affected both by local and by global distractor probability. This is indicated by the fact that in the test phase, when the distractor probability was set at the lowest level (6.6%) at each location, the amount of capture increased at all locations, including the previous low-probability location where the distractor maintained the same rate of occurrence of the training phase. If in Experiment 1 this pattern could be explained by invoking a general recovery of capture caused by the extinction phase, during which the distractor was removed, the same pattern of results was replicated also in Experiment 2, when no extinction phase was present, thus requiring a different explanation. Previous studies using a similar paradigm, and in which the distractor probabilities after training were equalized across the different locations, did not report the same finding, likely because in the test phase of such studies the distractor probability decreased at the previous high-probability location and increased at the previous low-probability location(s), which kept the overall distractor probability identical in the training and test phases (e.g., Ferrante et al., 2018; Wang & Theeuwes, 2020). By contrast, by equalizing in the distractor probabilities at the lowest level (of the training phase) during the test phase, we were able to reveal that the global distractor probability affects the degree of filtering applied at a specific distractor probability location. Our results thus indicate that the mechanism responsible for SL of distractor location takes into account the local and global distractor probability, and adjusts the level of local suppression accordingly. This reveals that the distractor filtering mechanism based on SL is more sophisticated than previously thought, being affected also by “contextual probabilistic information” concerning the overall distractor rate of occurrence. In fact, given the capacity of such mechanism to regulate the degree of filtering according to different distractor probabilities at distinct locations, it is likely that the amount of inhibition exerted locally could also be affected by the combined probabilities at all locations. In our view, the overall distractor probability sets the propensity of the cognitive system to activate more or less efficiently the filtering mechanism, or, to put it in signal detection theory terminology (Green & Sweets, 1966), the overall distractor probability (implicitly) makes the observer being more conservative or liberal in his or her suppressive strategy—namely, more or less prone to suppression—and this is irrespective of the specific rate of distractor occurrence at a given location. It follows that, for example, a location hosting the distractor on 10% of trials would be more actively suppressed when the overall distractor rate of occurrence is 70% rather than 30%. A similar idea, though not in relation to SL of distractor suppression, has been proposed by Müller and collaborators (2009; also see Geyer et al., 2008). The authors found that the amount of capture elicited by a distractor presented at a given rate (e.g., on 50% of the trials) differs as a function of whether the participants are previously exposed to the same distractor at a higher (e.g., 80%) or lower (e.g., 20%) rate. To explain the results, the authors proposed that when the rate of distractor occurrence is high, the filtering mechanism is robustly activated, thus reducing the amount of capture. By contrast, when the distractor rate is low, the mechanism is less active, yielding high distractor interference. Crucially, however, once activated at a given level, the filtering mechanism tends to remain in the same state of activation in future occasions in which the distractor is encountered. This would lead to different amounts of capture for the same distractor rate depending on the previous state of activation.

We have so far interpreted our findings as evidence of suppressive effects occurring at the saliency map level, but, in fact, recent evidence has shown that, under certain circumstances, distractor suppression takes place at the dimension (e.g., color) map level. For example, Zhang et al. (2019) have shown that when the color of the distractor is fixed relative to the nondistractor items, attentional capture is modulated by the distractor location probability, whereas the target-location effect disappears. The fact that target processing is not affected by SL of distractor location when the distractor is absent suggests that suppressive signals are implemented at the color-based map level (Zhang et al., 2019). In Experiments 1 and 2, the target-location effect was not completely reliable, and certainly was lacking in the extinction phase of Experiment 1, two facts that did not allow us to firmly establish whether in the present study distractor suppression took place at the saliency map level or at the dimension map level (Liesefeld & Müller, 2020). However, when in Experiment 3 the ratio of the distractor appearing at the high-probability and low-probability locations was increased to 21:1, a robust and reliable target-location effect emerged. Interestingly, such effect on target discrimination in distractor-absent trials persisted during the genuine extinction phase, whereas it vanished in the test phase, when the distractor was reintroduced, and its probability of occurrence was the same at all distractor locations. This pattern of results suggests some considerations. The fact that the target-location effect was present during a true extinction phase is a novel finding that is in agreement with the idea that the distractor salience is attenuated via suppressive signals applied to the corresponding location at the saliency map level (Ferrante et al., 2018; Wang & Theeuwes, 2018), and that the resulting pattern of activation in the map is temporarily maintained in a LTM representation.

It remains unclear, however, why in some occasions the suppressive signal leads to an attentional capture attenuation (the distractor-location effect), while it has no direct impact on target discrimination on distractor-absent trials (the target-location effect). Indeed, while some previous studies reported both effects (e.g., Wang & Theeuwes, 2018), others failed to find the target-location effect (e.g., Ferrante et al., 2018), a discrepancy that could be accounted for by methodological differences between the studies. A recent study by Lin et al. (2020) seems to indicate that a large probability ratio between the high-distractor versus low-distractor location is necessary to observe the target-location effect, an observation that is confirmed in our study. However, we also found that a dissociation between the distractor-location effect and the target-location effect emerged in the test phase, as with the same probability of distractor occurrence at each location one would have predicted the absence of both the target-location effect and the distractor-location effect. By contrast, whereas the former did not survive after the extinction phase, the latter was reliably still evident in the test phase.

As for the speed with which SL of distractor location is achieved, our combined analysis of the Experiment 1 and 2 training phase shows that this form of learning can emerge very rapidly, becoming evident after approximately 75 trials, when a reliable performance difference between the different distractor-probability locations is observed (see Fig. 9 and Table 1). This is much faster than what has been previously reported, like, for example, in the study of Ferrante et al. (2018), where SL of distractor location required at least 150 trials to become manifest. However, it should be acknowledged that the number of trials necessary for SL of distractor location to emerge depends on the overall probability of the distractor occurrence, and on the relative probability difference between the different distractor conditions. Indeed, an overall low-distractor probability provides the cognitive system with few occasions to learn the different distractor distributions, while a reduced difference between, for example, the high-probability and low-probability conditions clearly reduces the capacity of the system to detect and learn the different rates of distractor occurrence, and to use this information to adjust a proper suppressive bias accordingly.

A final issue concerns whether the reduction of capture obtained in this and other similar previous studies may have some relation to the habituation of capture (Turatto & Pascucci, 2016), which is a special case of the more general habituation of the orienting response (Sokolov, 1963; Waters et al., 1977). Indeed, in a number of studies we have recently shown that the attenuation of capture observed after repeated exposure to a visual distractor can be straightforwardly explained by invoking a habituation mechanism (Bonetti & Turatto, 2019; De Tommaso & Turatto, 2019; Pascucci & Turatto, 2015; Turatto et al., 2019; Turatto, Bonetti, & Pascucci, 2018a; Turatto, Bonetti, Pascucci, & Chelazzi, 2018b). Some common features between habituation of capture and SL of distractor location may suggest at least the existence of some links between the two phenomena. For example, both the effects of SL and habituation increase as the rate of stimulation (here, the probability of the distractor) increases. Secondly, usually SL of distractor location takes place implicitly (e.g., Wang & Theeuwes, 2018), without the need of top-down control (Duncan & Theeuwes, 2020). In a similar vein, habituation also occurs reflexively as the organism is repeatedly exposed to the same repetitive (usually irrelevant) stimulation (Sokolov et al., 2002; Steiner & Barry, 2014). Accordingly, we have shown that the habituation of attentional capture elicited by an irrelevant sudden onset emerges even when the irrelevant stimulus is presented in passive viewing (Turatto et al., 2018a; also see, Won & Geng, 2020). In other words, the mechanism recruited to filter the irrelevant sensory input operates quite automatically and independently from the need to shelter target processing from interference.

Despite such similarities, it remains however possible that SL and habituation are different and reflect distinct underlying learning processes, which both operate, together with other mechanisms (Chelazzi et al., 2019; Geng et al., 2019; van Moorselaar & Slagter, 2020), to attenuate the impact of salient, albeit irrelevant, distracting stimuli.

## Footnotes

1 Notice that this test included trials where the distractor appeared in the same location as in the preceding trial (see below).

2 We present here the analysis conducted by averaging Blocks 2 and 3 of the training phase, so to compare the same number of trials in both the training and test phase. However, a comparison between RTs of only the last block of training (Block 3) and the test phase (Blocks 6 & 7) would also be appropriate given that RTs did not seem to be approaching an asymptote by Block 3. In this experiment the overall pattern of statistical significance does not change whether one considers for comparison only Block 3 or the average of Blocks 2 and 3.

3 Limitedly to this specific comparison, the statistical test yields a different result if the training phase is defined only by Block 3 as compared with aggregating the data by Blocks 2 and 3. In this case, the increase in capture between training and test results to be significant: *t*(35) = 1.791, *p* = .041, *d* = .298.
